# Adsorption of Acetic Acid Vapors by Inorganic–Organic Nano Materials: Implications for the Inhibition of the “Vinegar Syndrome” in 20th Century Motion Picture Films

**DOI:** 10.3390/molecules30061348

**Published:** 2025-03-17

**Authors:** Francesca Porpora, Lorenzo Lisi, Emiliano Carretti, Carlotta D’Aleo, Marianna De Sanctis, Samuele Baldini, Luigi Dei

**Affiliations:** 1 Department of Chemistry “Ugo Schiff” & CSGI Consortium, University of Florence, Via Della Lastruccia 3-13, 50019 Sesto Fiorentino, Italy; francesca.porpora@unifi.it (F.P.); lore.lisi31@gmail.com (L.L.); carlotta.daleo@edu.unifi.it (C.D.); samuele.baldini@unifi.it (S.B.); 2 Centro Nazionale Delle Richerche—Istituto Nazionale di Ottica (CNR-INO), Largo E. Fermi 6, 50125 Firenze, Italy; 3 Film Restoration Laboratory “L’Immagine Ritrovata”, Via Riva di Reno 72, 40122 Bologna, Italy; marianna.desanctis@immagineritrovata.it

**Keywords:** xerogels, polyvinyl alcohol formaldehyde polymers, hydroxide, oxide, carbonate nanoparticles, cellulose acetate, “vinegar syndrome”, motion picture films conservation

## Abstract

Cellulose acetate (CA) motion picture films are subjected to degradation, especially due to the “vinegar syndrome”, a de-acetylation process catalyzed by high temperature, humidity, and acidity. Acetic acid is released as a by-product of this reaction and acts as a catalyst that triggers an autocatalytic process. The main aim of this study was to evaluate the use of metal oxide, hydroxide, and carbonate nanoparticles, as well as their composite inorganic–organic systems, for the adsorption of acetic acid and the inhibition of the deacetylation process. Various nanoparticles (Ca(OH)_2_, ZnO and CaCO_3_) were compared in terms of their ability to adsorb glacial acetic acid vapors through gravimetry analysis, Fourier Transform Infrared (FTIR) Spectroscopy, X-ray diffraction (XRD), and Thermogravimetric Analysis (TGA). The variation in the size and morphology of the nanoparticles was investigated via Scanning Electron Microscopy (SEM), too. Subsequently, the most promising nanoparticles (ZnO) were incorporated into composite organic–inorganic systems, made of Whatman paper (WP) and polyvinyl alcohol formaldehyde (PVF) xerogels, and their ability to adsorb acetic acid vapors was again evaluated. Finally, the performances of both the pure ZnO nanoparticles and the organic–inorganic composite systems as inhibitors of the “vinegar syndrome” were assessed on artificially degraded motion picture films using a specifically developed and validated multi-analytical protocol.

## 1. Introduction

Motion picture films are fascinating artistic objects with a complex multi-layer structure [1], typically comprising an emulsion layer made of gelatin and a support layer, usually composed of polymeric material. Concerning their support, between the 1920s and 1950s, cellulose acetate (CA) was introduced as a safer alternative to the more flammable and hazardous cellulose nitrate [1,2]. Unfortunately, this material also showed instability issues, and from the 1950s CA was replaced by more stable polyesters [1,2]. The “vinegar syndrome” is the common denomination used to describe the main degradation phenomenon affecting CA-based motion picture films, specifically the deacetylation of the CA backbone with the release of acetic acid as a by-product [3]. This process is catalyzed by high temperature, moisture, and acidity, and the acetic acid itself acts as a catalyst for the reaction, triggering an autocatalytic process [3]. Another collateral reaction of the “vinegar syndrome” is the depolymerization of the polymeric backbone [3]. Macroscopic manifestations of the “vinegar syndrome”, such as the deformation and the shrinkage of the films and the detachment of the emulsion layer from the support due to differential deformation (commonly referred to as “channeling”), can severely compromise the usability and the fruition of the films [1,4]. In the context of safeguarding these objects, it is important to prevent the occurrence and/or inhibit the evolution of the “vinegar syndrome”. Today, there are two main approaches available to deal with this issue: (i) to inhibit the kinetics of the reaction by acting on the regulation of the thermohygrometric conditions of the environments in which these objects are stored (e.g., by conserving the most degraded films at very low temperature and humidity values, according to the Guide published by Image Permance Institute [4]); (ii) to subtract the acetic acid in order to inhibit the autocatalytic process through its adsorption and/or neutralization. Concerning this last point, a lot of inhibitors have been tested in recent years, such as molecular sieves [5], activated charcoals [6,7], CaCO_3_ [7,8,9,10], Na_2_CO_3_, and sodium polyacrylate [11]), and in each case, there are some positive and negative aspects, as reported in the above-quoted literature. The most recent proposal concerns the use of water-stable Metal–Organic Frameworks (MOFs) [12,13,14], which seems the best option, but has the main drawback of requiring complex and expensive syntheses. 

In the last three decades, inorganic nanoparticles have been widely tested for the deacidification of cellulosic media of historical and artistic interest [15,16]; hydroxides of alkaline earth metals (such as calcium and magnesium hydroxides, Ca(OH)_2_ and Mg(OH)_2_) have been extensively used for the deacidification of paper, canvas, and archeological water-logged wood with excellent results. Part of the hydroxides are consumed for the neutralization of the acidity of cellulose, while the remaining particles react with CO_2_, turning into CaCO_3_ (Reaction (1)) which can act as a buffer against further acidity (Reaction (2)). Calcium hydroxide nanoparticles have been also tested for the adsorption of acetic acid vapors emitted by the wood of a church organ [17].

(R1) $${Ca\left(OH\right)}_{2}+2{CH}_{3}COOH\to {Ca\left({CH}_{3}COO\right)}_{2}+2H_{2}O$$

(R2) $${CaCO}_{3}+2{CH}_{3}COOH\to {Ca({CH}_{3}COO)}_{2}+H_{2}O+{CO}_{2}$$

Recently, a very interesting study was conducted on the use of ZnO nanoparticles loaded in castor oil polyurethane sponges and used for the adsorption of acetic acid [18,19]. The sponge is easy to handle, with no risk of dispersing particulate into the cases near the work of art (as in the case of gel silica or activated charcoal, for example). In addition, polyurethane acts as a vehicle for the gas through physisorption, and acetic acid can reach the surface of ZnO particles. ZnO is converted into zinc acetate, neutralizing the acetic acid (Reaction (3)). It was shown how the sizes of nanoparticles play an important role in the adsorption capacity of the system: decreasing the size of nanoparticles, the amount of the adsorbed acetic acid increases, even if the aggregation of the particles during the synthesis procedures can affect their performance. Nevertheless, while these systems can be used for the adsorption of acetic acid on large museum display cases, they seem not to be suitable for the adsorption of acetic acid in restricted volumes where they could be in direct contact with motion picture films. These systems being composed of biobased chemicals, due to the thermohygrometric conditions of the microenvironment that is formed in the motion picture films’ cases, they can promote the development of biological contamination.

(R3) $$2{CH}_{3}COOH+ZnO\to Zn{\left({CH}_{3}COO\right)}_{2}+H_{2}O$$

Thus far, the use of inorganic nanoparticles as a possible solution for the “vinegar syndrome” in the context of CA-based motion picture films has not been proposed and investigated. The main aim of this work was to test the performance of metal oxide, hydroxide, and carbonate nanoparticles, first in the adsorption of acetic acid vapors, and then in the inhibition of the deacetylation process in artificially degraded CA-based motion picture films. Various nanoparticles (Ca(OH)_2_, CaCO_3_/Ca(OH)_2_ mix, and ZnO) were compared in terms of their adsorption of glacial acetic acid vapors. Fourier Transform Infrared (FTIR) Spectroscopy, X-ray diffraction (XRD), and Thermogravimetric Analysis (TGA) were performed to qualitatively assess the occurrence of the acetate formation, and gravimetry tests were used to quantify the amount of acetic acid vapor adsorption. The variation in the morphology of the nanoparticles was investigated through Scanning Electron Microscopy (SEM) micrographs acquired before and after the exposition of acetic acid vapors.

Afterward, the more promising nanoparticles (ZnO) were included into composite organic–inorganic systems made of Whatman paper (WP) and polyvinyl alcohol formaldehyde (PVF) xerogels. This step is necessary in order to provide conservators and archivists with an easy-to-handle system. PVF-ZnO xerogels were characterized in terms of chemical composition (Thermogravimetric Analysis, TGA), and morphology (Scanning Electron Microscopy, SEM, and X-ray micro-tomography, μ-TOM). In addition, preliminary glacial acetic acid vapors adsorption tests were carried out on both composite WP- and PVF-based organic–inorganic systems.

Finally, the performances of pure ZnO nanoparticles and composite organic–inorganic systems in the inhibition of the “vinegar syndrome” on motion picture films in which the de-acetylation reaction was artificially induced with a proper degradation protocol [20] were evaluated. The different behaviors of films stored with and without the proposed inhibitors were tested through a specifically developed and previously validated multi-analytical protocol by considering the variation in free acidity, acetyl content (estimated via the Heterogeneous Saponification Metod and FTIR-ATR Spectroscopy) and tensile strength (tensile test) [20].

## 2. Results

### 2.1. Acetic Acid Adsorption–Desorption Tests on Nanoparticles

#### 2.1.1. Gravimetric Analysis

To evaluate the adsorption capacity of the selected nanoparticles (Ca(OH)_2_, CaCO_3_ mixed with Ca(OH)_2_, ZnO), they were subjected to an adsorption test of glacial acetic acid vapors. 

**Figure 1:**
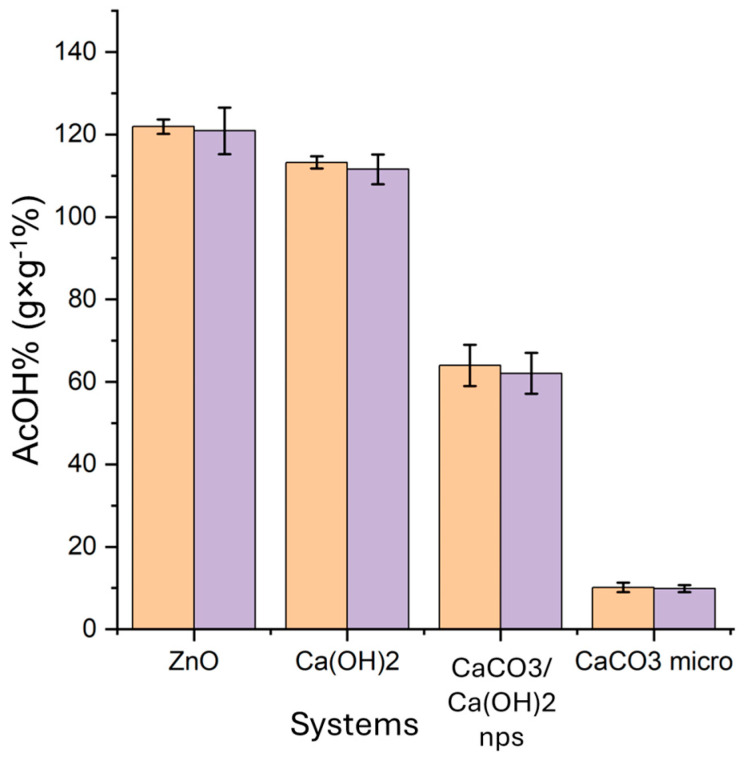
Adsorbed AcOH_ad_% after the adsorption test (Equation (3), orange) and after the desorption test AcOH_convert._% (Equation (4), purple) for Ca(OH)_2_, CaCO_3_/Ca(OH)_2_, ZnO nanoparticles, and CaCO_3_ micropowder.

In Table 1 and Figure 1, the increase in weight calculated after six days of exposition to acetic acid vapors and six days of equilibration (AcOH_ad_%, Equation (3) in Section 4, column 1 of Table 1) and after the desorption test (AcOH_convert._%, Equation (4) in Section 4, column 2 of Table 1) are reported for each kind of nanoparticle and the CaCO_3_ micropowder for comparison. In addition, the last two columns of Table 1 report the amount of the residual adsorbed acetic acid not converted into acetate (AcOH_resid._%, Equation (5) in Section 4, column 3 of Table 1), and the theoretical increase in weight expected from the entire conversion of the starting nanoparticles to the corresponding acetate salt (column 4 of Table 1) is calculated as follows:

(1) $${AcOH}_{T}\%=\frac{{Mw}_{Ac\;salt}-{Mw}_{0}}{{Mw}_{0}}\;*\;100$$

in which *Mw*_0_ is the molecular weight of the initial compound and *Mw*_*Acsalt*_ is the molecular weight of the corresponding acetate salt. 

The registered increase in weight could be associated with the almost total conversion of the compounds to the corresponding acetate salts. The very small amount of residual, non-desorbed acetic acid was derived from the adsorbed acetic acid that did not react with acetate salts. Indeed, the data reported in Table 1 show that almost the whole acetic acid vapor was converted into acetate that could not be desorbed under vacuum. These findings have been confirmed by FTIR, XRD and TGA analyses.

**Figure 2:**
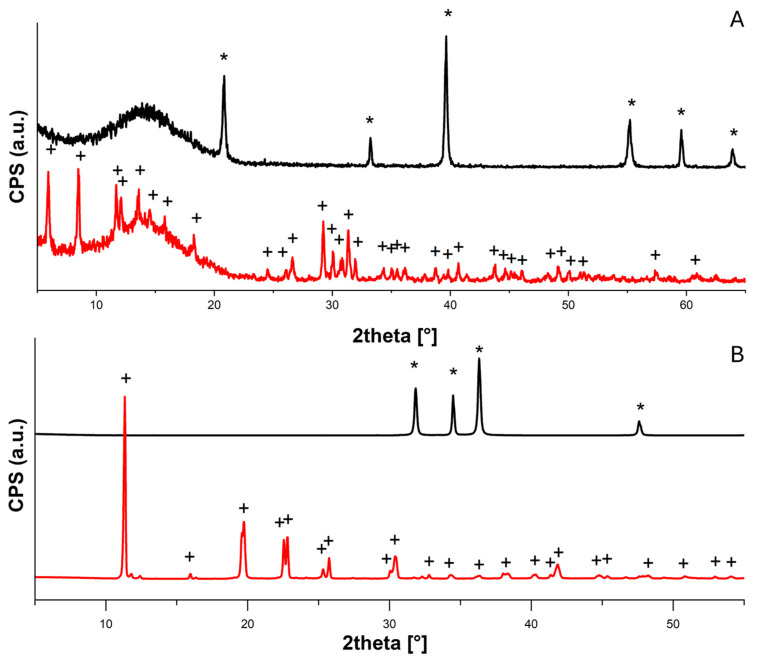
XRD diffractograms collected for (**A**) Ca(OH)_2_ nanoparticles before (black) and after (red) acetic acid adsorption test, peaks assigned to calcium hydroxide (portlandite, PDF 44-1481) are labeled with “*”, and to calcium acetate hydrate (PDF 19-0199) with “+”; (**B**) ZnO nanoparticles before (black) and after (red) acetic acid adsorption test, peaks assigned to zinc oxide (zincite PDF 04-020-0364) are labeled with “*” and to zinc acetate (PDF 02-064-1515) with “+”.

**Figure 3:**
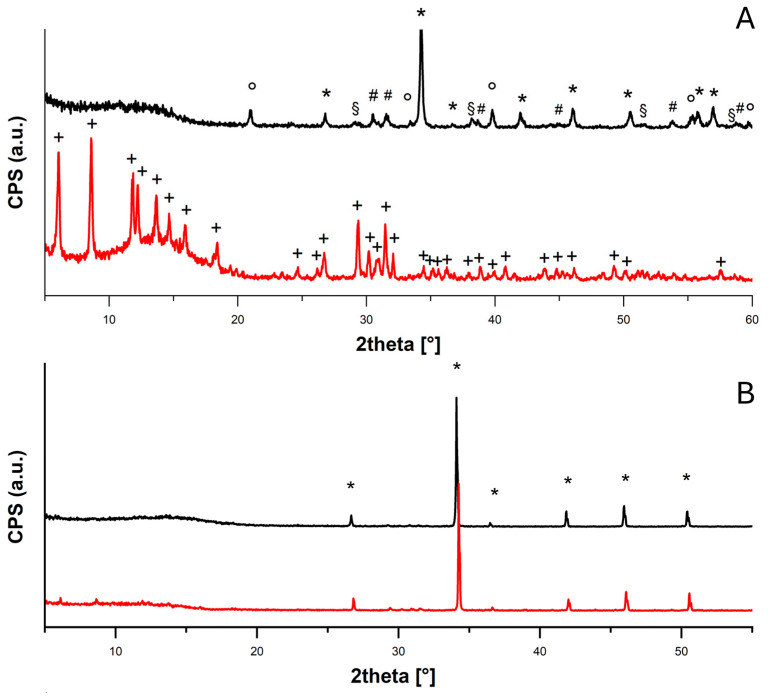
XRD diffractograms collected for (**A**) CaCO_3_/Ca(OH)_2_ nanoparticles before (black) and after (red) acetic acid adsorption tests; peaks assigned to calcite (PDF 83-0578), portlandite (PDF 44-1481), aragonite (PDF 75-2230) and vaterite (PDF 24-0030) are labeled with “*”, “°”, “#” and “§”, respectively, and to calcium acetate (PDF 19-0199) with “+”; (**B**) CaCO_3_ micropowder before (black) and after (red) acetic acid adsorption test, peaks assigned to calcite (PDF 83-0578) are labeled with “*”.

From the FTIR spectra (Figure S1 in Section S1.1) and XRD diffractograms (Figure 2 and Figure 3), it is possible to confirm the formation of the acetate salts from all the examined compounds.

#### 2.1.2. XRD Analysis 

Ca(OH)_2_ diffractograms acquired before and after the acetic acid adsorption test are reported in Figure 2A. The conversion from calcium hydroxide (portlandite, PDF 44-1481) to calcium acetate hydrate (PDF 19-0199) is evident. 

In Figure 2B, the formation of zinc acetate (PDF^®^02-064-1515) from pure ZnO (zincite PDF^®^04-020-0364) is clear, as shown when comparing the diffractograms acquired before and after the acetic acid adsorption test. 

From the first diffractogram shown in Figure 3A, it is possible to confirm the carbonatation of the calcium hydroxide nanoparticles into different polymorphisms of calcium carbonate—calcite (PDF 83-0578), aragonite (PDF 75-2230) and vaterite (PDF 24-0030)—with some residues of calcium hydroxide (portlandite, PDF 44-1481). These were probably due to the superficial carbonatation of the particles with the consequent isolation of the calcium hydroxide core, which was no longer accessible to CO_2_. Therefore, the experiments were carried out on a mixture of CaCO_3_/Ca(OH)_2_ nanoparticles. The conversion into calcium acetate hydrate (PDF 19-0199) after acetic acid exposure was also appreciable. On the contrary, the diffractograms registered for the CaCO_3_ micropowder both before and after the acetic acid adsorption test (Figure 3B) show the presence of only calcite (PDF 83-0578), in agreement with the very low conversion (<10%). 

#### 2.1.3. TGA Analysis

**Figure 4:**
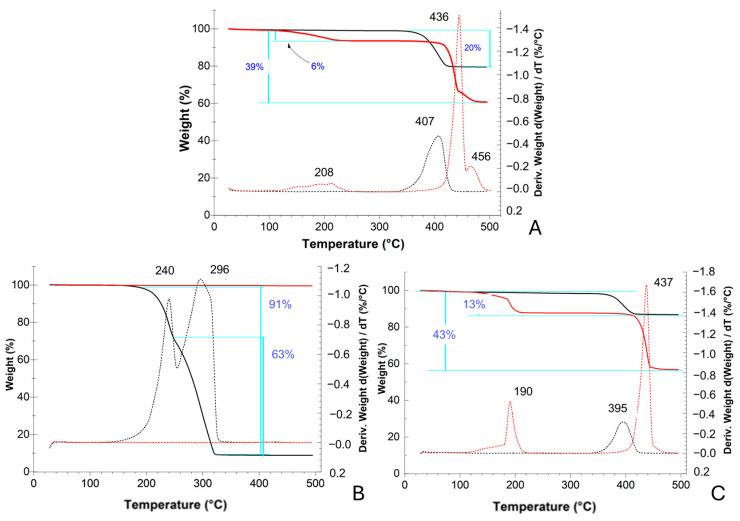
TGA (solid line) and DTG (dashed line) profiles of (**A**) Ca(OH)_2_ nps, (**B**) ZnO nps and (**C**) CaCO_3_ nps before (black) and after (red) the adsorption test in glacial acetic acid.

From the TGA and DTG profiles, further information on the nature of our systems and the reaction that occurred during the acetic acid adsorption test can be acquired (Figure 4). In particular, weight losses associated with decomposition reactions and calculated from TGA and DTG profiles can be compared to the theoretical weight losses calculated for those transitions in terms of variation in the molecular weights of the involved compounds,

(2) $$∆{Mw}_{t}=\frac{{Mw}_{B}-{Mw}_{A}}{{Mw}_{A}}\cdot 100$$

Here, *Mw_A_* is the molecular weight of the reagent and *Mw_B_* is the molecular weight of the product after the examined reaction.

For Ca(OH)_2_, in the TGA and DTG profiles (Figure 4A), a weight loss of 20% can be observed between 350 and 420 °C (peak in DTG at 407 °C) corresponding to the decomposition temperature of the calcium hydroxide into CaO, mainly due to water release (theoretical weight loss increment,$\;∆{Mw}_{t}$: 23%) [21,22]. In the profile of Ca(OH)_2__AcOH, collected after the adsorption period, a first weight loss of 6% can be observed between 100 and 230 °C (peak at 208 °C), and a second weight loss of 33% between 400 and 480 °C (peak at 435 °C). The first one could be ascribed to an initial mass loss of water molecules, while the second one to the breakdown of the dehydrated calcium acetate into acetone and calcium carbonate ($∆{Mw}_{t}$: 37%) [23]. The peak observed at 455 °C is likely attributable to the eventual loss of acetone. The further decomposition of calcium carbonate is not seen in this profile because it occurs at higher temperatures [24]. 

In the TGA ZnO profile (Figure 4B), no weight loss can be detected, as expected, because the thermal degradation of zinc oxide occurs at temperatures above 500 °C [25]. In the ZnO_AcOH profile, a total weight loss of 91% between 160 and 330 °C can be observed, and this corresponds, in the first range (160–250 °C, ~28%), to the loss of molecular water and adsorbed acetic acid, and in the second range (250–330 °C), to the thermal degradation of zinc acetate into zinc oxide (~46%), the sublimation of zinc acetate species, or the formation of other volatile zinc organic compositions such as Zn_4_O(CH_3_CO_2_)_6_ (residual ~17%) [26,27].

In the CaCO_3_ TGA profile (Figure 4C), a weight loss of 13% is visible between 340 and 420 °C (peak at 395 °C), due to the presence of residual calcium hydroxide in the starting sample (see diffractogram in Figure 3A). CaCO_3__AcOH’s TGA profile shows two weight losses, of 12% between 100 and 222 °C (peak at 190 °C) due to the removal of water molecules and 31% between 391 and 460 °C (peak at 437 °C) due to the thermal degradation of calcium acetate into calcium carbonate (theoretical weight loss: 37%) [22].

#### 2.1.4. SEM Micrographs 

**Figure 5:**
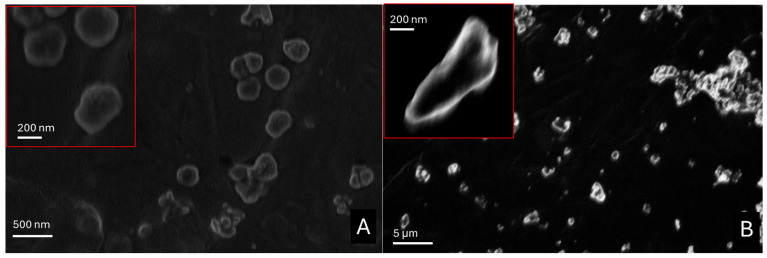
SEM micrographs acquired for ZnO nanoparticles before (**A**) and after (**B**) the acetic acid adsorption test.

SEM micrographs acquired for each compound before and after exposure to acetic acid vapors are reported below (Figure 5) and in the Supporting Information (Figure S2 in Section S1.2). 

In Figure S2A, hexagonal platelets with hexagon sides of 100 ± 50 nm and a thickness of 2–40 nm typical of portlandite are shown [28]. After the exposure to acetic acid, calcium acetate (Figure S2B) appeared as aggregates with a needle-like shape [29] from a few hundred nanometers to a few micrometers, compacted in a sort of cement. 

Zinc oxide appeared as pseudo-circular nanoparticles with sizes of approximately 100 nm (Figure 5A). After the adsorption test, the sizes of nanoparticles only slightly varied, but the presence of bigger aggregates was appreciated (Figure 5B). For zinc-based nanoparticles before and after the acetic acid adsorption test, a dimension analysis was performed with Image J software (v. 1.54). By approximating all the nanoparticles as rounded objects, zinc oxide nanoparticles showed a calculated radius of 140 ± 80 nm, while zinc acetate showed a size distribution of 180 ± 90 nm. 

After the carbonatation step (Figure S2C), calcium carbonate nanoparticles assumed an irregular pseudo-circular shape. Single nanoparticles did not noticeably vary their dimensions compared to calcium hydroxide nanoparticles (150 ± 90 nm), but several, bigger aggregates were evident (even up to 0.5–1 μm in diameter). In this case, for calcium acetate crystals obtained after the exposure to acetic acid vapors, the morphology and the dimension seemed not to sensibly vary from the initial ones (190 ± 100 nm), except for the presence of even bigger aggregates (Figure S2D).

On the other hand, the calcium carbonate micropowder showed a rhombohedral habit, typical of calcite, both before and after acetic acid adsorption (particle size was around 2–4 μm for the rhombus side, Figure S2E–F), which coheres with previous considerations.

### 2.2. Acetic Acid Adsorption–Desorption Tests on WP-ZnO and PVF-ZnO

Whatman paper (WP) and polyvinyl alcohol formaldehyde (PVF) xerogels were loaded with ZnO nanoparticles (WP-ZnO and PVF-ZnO, respectively), since ZnO revealed the best results in terms of acetic acid vapor adsorption, and were subjected to an adsorption–desorption test of acetic acid vapors. The physicochemical and morphological characterization of PVF-ZnO xerogels is reported in Section S2 of the SI.

#### 2.2.1. Gravimetric Analysis 

**Figure 6:**
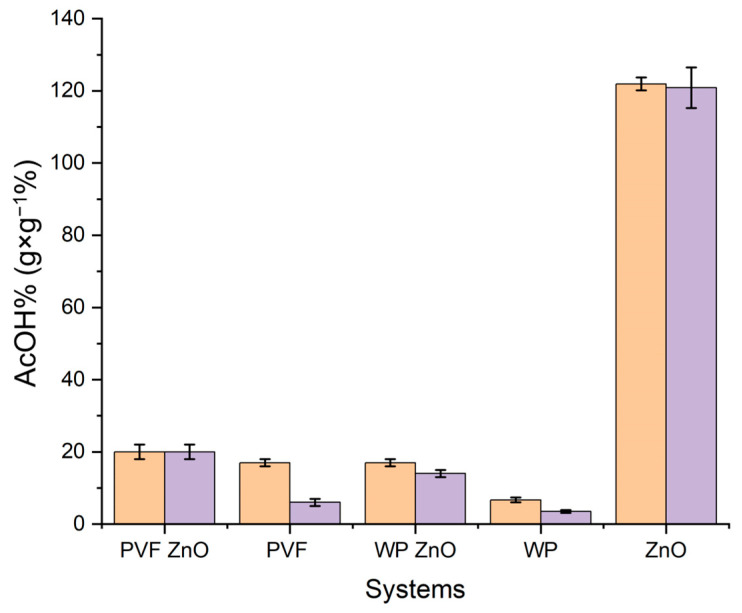
Adsorbed AcOH_ad_% after the adsorption test (6 days, Equation (3), orange) and after the desorption test AcOH_convert_.% (48 h under vacuum, Equation (4), purple) for PVF + ZnO, pure PVF, WP + ZnO, pure WP and ZnO nanoparticles.

Gravimetric analyses were performed, and AcOH_ad_% (Equation (3)), AcOH_convert._% (Equation (4)), and AcOH_resid._% (Equation (5)) were calculated and are reported in Table 2 and Figure 6. Even though we continue to use the terms AcOH_convert._ and AcOH_resid._, the meanings are completely different for the samples PVF and WP, since there was no conversion to acetate due to the lack of the metal oxide. In these two cases, AcOH_convert._ was the adsorbed acetic acid strongly bound to the support (WP or PVF) due to chemisorption, and AcOH_resid._ was the acetic acid weakly bound to the support due to physisorption. In the case of the mixed composite of WP/ZnO and PVF/ZnO, we have to consider three kinds of interactions, as follows: the interaction between gaseous acetic acid and the surface of nanoparticles, which allows acetic acid neutralization (AcOH_convert_); the weak, physical interaction between acetic acid and the organic composite of the system (WP or PVF) due to the entrapment of gaseous acetic acid inside the porosities of the systems (AcOH_resid_);

The strong chemisorption of the acetic acid into the organic materials, probably due to the action of the hydrogen bonds between free hydroxyl groups present in both PVF and WP (AcOH_convert_).

Therefore, AcOH_resid_ and AcOH_convert_ contained both acetic acid converted to acetate and residual acetic acid adsorbed and chemisorbed/physisorbed by WP and PVF.

The AcOH_ad_% (Equation (3)) after six days of adsorption and six of equilibration was only 6.7 ± 0.5% for pure WP, 17 ± 1% for WP-ZnO, 17 ± 1% for pure PVF and 27 ± 2% for PVF-ZnO. After the desorption test, AcOH_convert._% (Equation (4)) decreased for all the inorganic–organic systems (WP + ZnO—14 ± 1% and PVF + ZnO—20 ± 2%), and an analogous decrease was observed for the pure supports WP and PVF. The weight loss detected after the desorption test (AcOH_resid._%, Equation (5)) in all the samples was due to the acetic acid physisorbed on the paper or the xerogel. The strong difference concerning pure ZnO nanoparticles was due to the smaller amount of ZnO in the composite systems, which contained approximately 10 *w*/*w*% of ZnO. 

#### 2.2.2. FTIR-ATR Analysis

**Figure 7:**
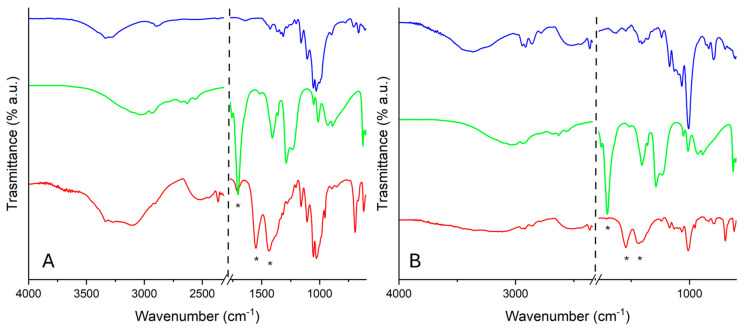
FTIR-ATR spectra registered on WP-based (**A**) and PVF-based systems (**B**) uploaded with ZnO nanoparticles before (blue) and after (red) the adsorption test with glacial acetic acid vapors and the spectrum of glacial acetic acid (green). Peaks associated with the formation of zinc acetate are labeled with “*”.

In Figure 7, FTIR-ATR spectra of WP (Figure 7A) and PVF (Figure 7B) uploaded with ZnO nanoparticles before and after the adsorption test with glacial acetic acid vapors are reported. It is possible to appreciate the peaks ascribable to zinc acetate—C–O asymmetric and symmetric stretching vibrations modes are detected at about 1550 and 1435 cm^−1^, respectively [18,30]. In addition, a peak at about 1700 cm^−1^ is detected, ascribable to the asymmetric stretching of the C=O group.

#### 2.2.3. TGA

**Figure 8:**
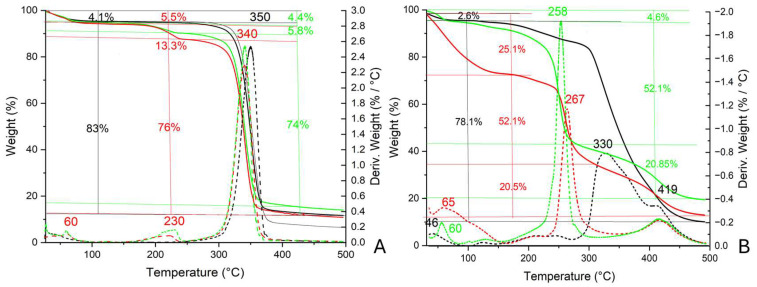
TGA (solid line) and DTG (dashed line) profiles of WP-ZnO (**A**) and PVF-ZnO (**B**) before the adsorption test (black), after the adsorption test of acetic acid vapors (red), and after the desorption test (green).

In Figure 8, TGA and DTG profiles associated with WP-ZnO and PVF-ZnO after the acetic acid adsorption and desorption test are reported.

Concerning WP-ZnO (Figure 8A), the first weight loss of 4.1% at about 60 °C was associated with adsorbed moisture. The weight loss of 83% between 300 and 370 °C (peak at 350 °C in the DTG profile) is ascribable to the decomposition of the Whatman paper. In the thermogravimetric profile of WP-ZnO after the adsorption of acetic acid, the first weight loss at ca. 60 °C was about 5.5%; it was only slightly higher than the one calculated for pure WP-ZnO, probably because the amount of weakly adsorbed acetic acid was very low. An additional weight loss of about 13.3% was registered between 150 and 250 °C (peak at 232 °C of the DTG profile), and it is possible to ascribe this to the decomposition of zinc acetate (see ZnO-AcOH profile in Figure 4B). Finally, the last weight loss between 250 and 360 °C (peak at 340 °C) was associated with the thermal decomposition of the Whatman paper. 

In the profile associated with PVF-ZnO (Figure 8B) after the adsorption test, a first weight loss of 25.1% was observed between 35 and 180 °C, and could be ascribed to the loss of moisture and acetic acid weakly bonded to the xerogel (peak at 65 °C in the DTG profile); the second weight loss between 180 and 350 °C (peak at 267 °C) was due to the thermal degradation of zinc acetate inside the xerogel (the same weight loss of 63% is visible for ZnO-AcOH, see Figure 4B). A last weight loss was registered between 350 and 500 °C (peak at 419 °C), and this could be associated with the degradation of PVF (see Figure S3 in Section S2.2.1 of the Supporting Information). Concerning PVF-ZnO after the desorption test, the first weight loss ascribable to the loss of moisture and weakly bonded acetic acid was lower than that immediately after the adsorption test (of 4.6% vs. 25.1%) and occurred at lower temperatures (between 44 and 90 °C). The weight loss of 52.1% between 150 and 350 °C (peak at 258 °C) could also be ascribed to the presence of zinc acetate inside the xerogel, and the second weight loss of 20.85% between 350 and 500 °C (peak at 419 °C) could be ascribed to the degradation of the PVF sponge.

#### 2.2.4. SEM Micrographs

**Figure 9:**
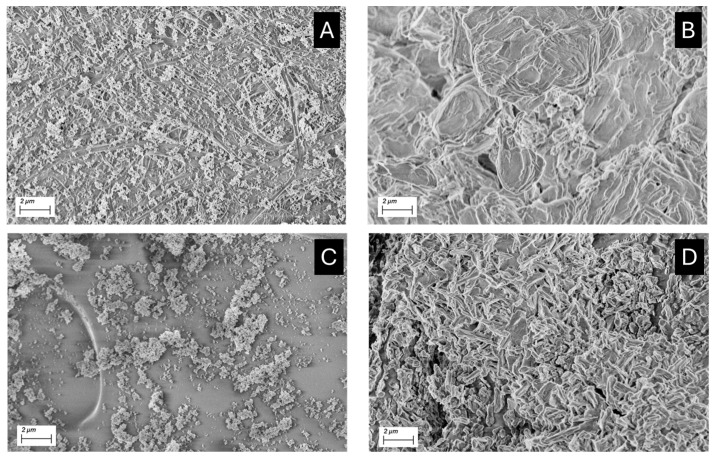
Whatman paper soaked with ZnO nanoparticles (WP-ZnO) before (**A**) and after (**B**) the exposure to acetic acid vapors; PVF xerogels uploaded with ZnO nanoparticles (PVF-ZnO) before (**C**) and after (**D**) the exposure to acetic acid vapors.

In Figure 9, SEM micrographs acquired for both WP-ZnO and PVF-ZnO before and after the acetic acid adsorption test are shown. 

For both WP-ZnO (Figure 9A) and PVF-ZnO (Figure 9C), it is possible to see a very dense layer of ZnO nanoparticles on the surfaces of the systems. In particular, ZnO nanoparticles seem to be trapped between the fibers of the Whatman paper, and in some porosities and/or on the surface of PVF xerogels, forming some bigger aggregates.

After the adsorption of acetic acid (Figure 9B,D), a compact layer became visible on the surfaces of both systems (Figure 9D). 

### 2.3. The Evaluation of the Performance of ZnO Pure Nanoparticles, WP-Zno and PVF-Zno Xerogels on Artificially Degraded Real Motion Picture Films

All the samples of real motion picture films (RMPF) subjected to the ATM2.9 degradation protocol are also depicted in Table 3. 

#### 2.3.1. Free Acidity

**Figure 10:**
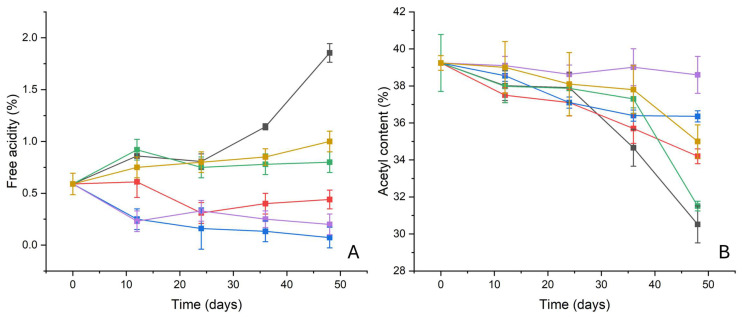
(**A**) Free acidity and (**B**) correct acetyl content calculated for RMPF, on which the deacetylation process was triggered by expsore to a saturated atmosphere of HCl (0-point of the graph) and then stored for 12, 24, 36 and 48 days at 100% RH without any inhibitor (PX_ATM2.9_NT, black), with pure PVF (PX_ATM2.9_PVF, green), with pure WP (PX_ATM2.9_WP, yellow), with PVF-ZnO (PX_ATM2.9_PVF-ZnO, purple), with WP-ZnO (PX_ATM2.9_WP-ZnO, blue), and with ZnO nps (PX_ATM2.9_ZnOnps, red). The results of each measurement are expressed as the average value and the corresponding standard deviation calculated for three fragments of the same film subjected to the same experiment.

The trend of the free acidity reported in Figure 10A suggests very interesting results; for samples subjected to the ATM2.9 degradation protocol without any inhibitor (NT), free acidity values increased by almost 200% (from ca 0.6 ± 0.07% for the sample P9_HCl5M up to 1.85 ± 0.09% for the sample P48_ATM2.9_NT) [20]. On the other hand, for samples degraded in the presence of pure ZnO nanoparticles, the free acidity decreased after the 24th day (P24_ATM2.9_ZnOnps—0.3 ± 0.1%), but it increased again until the 48th day (P48_ATM2.9_ZnO nps—0.44 ± 0.09%). For samples degraded in the presence of pure PVF, higher free acidity values were detected for the entire duration of the degradation protocol, at ca. 0.8%, even if a sort of stabilization was observed around this value. Similar values of free acidity were detected for film stored with pure WP, even if an increase in the free acidity was monitored for the entire duration of the test (P48_ATM2.9_WP—1.1 ± 0.1%). On the contrary, samples degraded in the presence of both organic–inorganic systems showed very promising results—a stabilization of the free acidity was detected for samples degraded in the presence of Whatman^®^ (Maidstone, UK) paper doped with ZnO nanoparticles, and after 12 days, the free acidity decreased from 0.6 ± 0.1% to ca 0.25 ± 0.1%. The free acidity values continued decreasing for the entire duration of the degradation protocol, ranging between 0.3% and 0.1%.

Also, for samples subjected to the ATM2.9 degradation protocol in the presence of PVF + ZnO, a decrease in the free acidity was observed in the first 12 days (P9_HCl5M—0.60 ± 0.09% vs. P12_ATM2.9_PVF + ZnO—0.2 ± 0.1%), with a subsequent stabilization that lasted until the end of the test (P48_ATM2.9_PVF + ZnO—0.2 ± 0.1%). 

#### 2.3.2. Acetyl Content

The initial acetyl content of the motion picture films used for the test was 41.7 ± 0.7%, typical of a cellulose diacetate [3,31]. After the first phase of the degradation protocol (exposure to HCl vapors for 9 days), the deacetylation process was triggered, with a consequent decrease in the acetyl content to 39.2 ± 0.4% (Figure 10B). 

A significant decrease (almost 23%) was observed not only for the NT samples (P48_ATM2.9_NT—30 ± 1), but also for the samples degraded at 100% RH in the presence of pure ZnO nanoparticles, pure PVF and pure WP, even if this effect was less pronounced in these last cases (the decrease in the acetyl content after 48 days was almost 10%, 13% and 20% for samples degraded in presence of pure WP, ZnO nanoparticles and pure PVF, respectively). The decrease in the acetyl content started between the 24th and the 36th days for samples stored with no inhibitor (P36_ATM2.9_NT—36 ± 1%) and with ZnO nanoparticles (P36_ATM2.9_ZnOnps—34.2 ± 0.4%), and after the 36th day for RMPF stored with pure WP (P48_ATM2.9_WP—35.0 ± 0.9%) and pure PVF (P48_ATM2.9_PVF—31.5 ± 0.3%).

On the contrary, the trend in the acetyl content was strongly influenced by the presence of both the organic–inorganic systems; the Whatman paper doped with ZnO nanoparticles induced a stabilization of the acetyl content after 24 days of the degradation protocol, at about 37% (for samples P24_ATM2.9_WPZnO, the acetyl content value was 37.1 ± 0.3%). A similar trend was also observed in the correct acetyl content for RMPF degraded in the presence of PVF + ZnO, which remained almost constant for the entire duration of the degradation process (P9_HCl5M—39.2 ± 0.4%; vs. P48_ATM2.9_PVF + ZnO—38 ± 1%).

#### 2.3.3. FTIR-ATR Spectroscopy 

**Figure 11:**
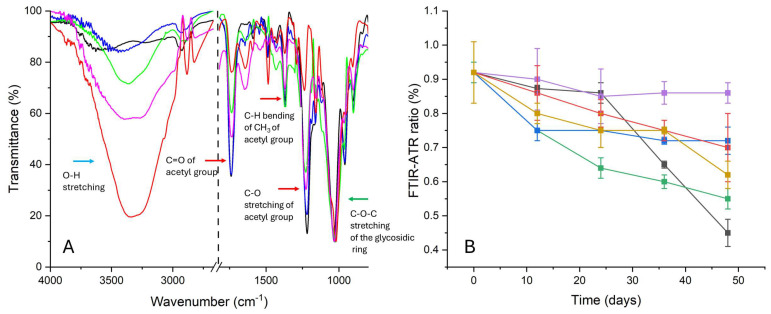
(**A**) FTIR-ATR spectra of P0 (black), P9_HCl5M (blue), P48_ATM2.9_NT (red), P48_ATM2.9_PVF-ZnO (green), P48_ATM2.9_WP-ZnO (magenta); (**B**) FTIR-ATR ratio calculated between the peaks at 1220 and 1030 cm-1 for PX_ATM2.9_NT (black), PX_ATM2.9_WP (yellow), PX_ATM2.9_PVF (green), PX_ATM2.9_PVF-ZnO (purple), PX_ATM2.9_WP_ZnO (blue) and PX_ATM2.9_ZnOnps (red). The results of each measurement are expressed as the average values and the corresponding standard deviations calculated from five spectra acquired in different areas of the film subjected to the same experiment.

From the FTIR-ATR spectra (Figure 11A), it is possible to obtain information about the deacetylation process in a non-invasive and non-destructive way [32,33,34,35]; for films degraded in the absence of inhibitors, after the 24th day, a decrease in the intensity of the peaks associated with the acetyl group (peak at 1220 cm^−1^ of the C–O stretching, at 1330 cm^−1^ of the C–H bending and peak at 1730 cm^−1^ of the C=O stretching) and an increase in the intensity of the peak ascribed to the O–H stretching of the hydroxyl group at ca. 3330 cm^−1^ were observed if compared to the peak at 1030 cm^−1^ of the C–O–C stretching of the glycosidic ring, used as the internal standard. This trend was measured for the entire duration of the degradation protocol. Consequently, the ratio between the intensity peaks at 1220 cm^−1^ and 1030 cm^−1^ (Figure 11B) slowed down from 0.92 ± 0.08 for P9_HCl5M to 0.45 ± 0.04 for P48_ATM2.9_NT, confirming the artificial induction of the deacetylation process.

For samples degraded in the presence of pure PVF and pure WP, the trend was very similar to that registered for NT motion picture films: a strong decrease in the I_1220_/I_1030_ ratio was observed already after the 12th day, with a significant diminuition after 48 days, as indicated in Figure 11B (the I_1220_/I_1030_ ratio is 0.55 ± 0.03 for P48_ATM2.9_PVF and 0.62 ± 0.03 for P48_ATM2.9_WP). Even if the intensities of the peaks due to the acetyl group (peaks at 1220, 1330 and 1730 cm^−1^) after 48 days of duration of the degradation protocol were more intense in samples degraded with ZnO nanoparticles (P48_ATM2.9_ZnO nps) than in the NT ones, the ratio between the intensities of the peaks at 1220 cm^−1^ and at 1030 cm^−1^ up until the 36th day decreased (P9_HCl5M—0.92 ± 0.09; P36_ATM2.9_NT—0.65 ± 0.1 vs. P36_ATM2.9_ZnO nps—0.75 ± 0.03), and a slowing of the degradation process was observed between the 36th and 48th days (P48_ATM2.9_NT—0.45 ± 0.04; P48_ATM2.9_ZnO nps—0.7 ± 0.1%). 

In accordance with previous tests, the behavior of RMPF stored with organic–inorganic systems was also deemed promising according to the FTIR-ATR analysis. For all the motion picture films degraded in the presence of WP-ZnO and PVF-ZnO, the ATR-FTIR spectra indicate that the decrease in the intensities of the peaks associated with the acetyl group (peaks at 1220, 1330 and 1730 cm^−1^) was very low for all durations of the application of the ATM2.9 protocol. Even for the intensity of the peak associated with the OH group, only a low increase was observed for all the treated samples after 48 days, probably due to the adsorption of moisture. Indeed, no significative variations in the I_1220_/I_1030_ ratio were detected for the samples degraded in the presence of the PVF xerogels containing the nanoparticles (P48_ATM2.9_PVF-ZnO: 0.86 ± 0.03). For RMPF degraded in the presence of WP-ZnO after 12, days a low decrease was observed (from 0.92 ± 0.09 for P9_HCl5M to 0.9 ± 0.03 for P12_ATM2.9_WP-ZnO), with a subsequent stabilization around this value up to the 48th day of the degradation process (the I_1220_/I_1030_ ratio is 0.72 ± 0.04 for P48_ATM2.9_WP-ZnO). 

#### 2.3.4. Tensile Tests 

**Figure 12:**
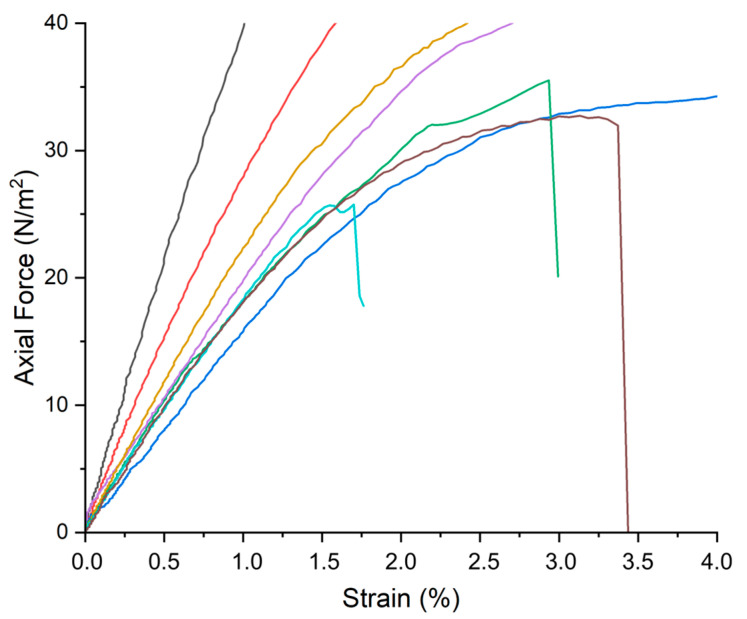
Axial force (N/m^2^) versus strain graph registered for RMPF samples exposed to the ATM2.9 degradation protocol at different days of exposure and with or without the inhibitors: P0 (black), P9_HCl5M (red), P48_ATM2.9_NT (blue), P48_ATM2.9_WP-ZnO (yellow), P48_ATM2.9_PVF-ZnO (purple), P48_ATM2.9_PVF (green), P48_ATM2.9_WP (brown), P48_ATM2.9_ZnO nps (cyan). For each sample, five replications were are performed; in the figure, one of the five replications is reported as an example for each analyzed sample.

Variations in tensile strength during the ATM2.9 degradation protocol were investigated with tensile tests, and the results are reported in Figure 12 (calculating the slope of the axial force vs. strain curve allowed us to obtain the Young’s modulus). A greater decrease in the tensile strength was observed for RMPF stored without any inhibitor—a reduction in the Young’s modulus by half was registered between P9_HCl5M (14 ± 1 MPa) and P48_ATM2.9_NT (7.3 ± 0.2 MPa).

On the other hand, for samples stored with pure PVF, pure WP, PVF-ZnO and pure ZnO nanoparticles (9 ± 2 MPa), and WP-ZnO (10 ± 2 MPa) for 48 days of the ATM2.9 degradation protocol, a slight decrease in the Young’s modulus was observed, but this was lower if compared to NT samples. 

In addition, it was interesting to note that, while for RMPF stored with WP-ZnO and PVF-ZnO and without any inhibitor, a plastic deformation before 40 N was observed, three of the five samples stored with ZnO nps and four of the five samples stored with pure WP and PVF broke at 25 ± 2, 3 ± 2 and 35 ± 3 N, respectively.

## 3. Discussion

### 3.1. Acetic Acid Adsorption–Desorption Tests on Nanoparticles and Composed Organic–Inorganic Systems

In this work, the possibility of using nanoparticle-based systems for the inhibition of the “vinegar syndrome” was evaluated. 

First of all, we tested the acetic acid vapor adsorption capacities of various nanoparticles (Ca(OH)_2_, CaCO_3_/Ca(OH)_2_, ZnO); all the systems showed good performance in terms of the total adsorbed acetic acid (AcOH_ad_%, Equation (3)), and the conversion to acetate salts was practically total (AcOH_convert._%, Equation (4))—ZnO, 121 ± 3% and 120 ± 6%; Ca(OH)_2_, 113 ± 2% and 111 ± 4%; CaCO_3_/Ca(OH)_2_, 64 ± 2% and 62 ± 5% (see Table 1 and Figure 1). The capacity to strongly bind the adsorbed acetic acid by converting it into the corresponding acetate salt was confirmed by FTIR (Figure S1), XRD (Figure 2 and Figure 3) and TGA analyses (Figure 4). 

On the other hand, the AcOH_ad_% of CaCO_3_ micropowder (10 ± 1%, Table 1) was noticeably lower than that of the corresponding nanosized CaCO_3_ mixed with Ca(OH)_2_ (64 ± 2%, Table 1), likely due to the lower capacity of the micropowder to adsorb acetic acid with respect to the mixture of CaCO_3_ and Ca(OH)_2_ nanoparticles having a high specific surface area. The diffractograms also confirm that the reaction between the acetic acid vapors and the metal oxide/hydroxide/carbonate that produced acetate salts occurred only for nanoparticles, and not for the micropowder (for CaCO_3_ micropowder, only peaks ascribable to calcite are visible in the diffractogram after the acetic acid adsorption test; see Figure 3B), implying the indispensability of using nanomaterials. It is worthwhile to mention that the present results confirm what had already been determined about CaCO_3_ micropowder in previous works [10,36], i.e., mediocre results in terms of the inhibition of the “vinegar syndrome” and the prevention of the infection of other films stored nearby. Our results are coherent with those of these studies, and confirm the great advantage offered by the use of nano-systems instead of massive materials; it is well-known that the decrease in size corresponds to a decrease in the number of atoms that form the particle. Moreover, the smaller the particles, the greater the number of surface atoms, with a consequent improvement in several properties, such as the chemical reactivity [15]. 

Between the different kinds of examined nanoparticles, a greater increase in weight was calculated for ZnO nanoparticles, so ZnO was chosen to perform further experiments on its application in real motion picture films affected by the “vinegar syndrome”.

When comparing pure WP and PVF with the corresponding systems loaded with ZnO nanoparticles, it was evident that the latter showed higher AcOH_ad_% values (Table 2, Figure 6). Considering that the amount of ZnO nanoparticles loaded within the support (WP or PVF) was approximately 10 *w*/*w*%, and looking at Table 2 and Figure 6, it is possible to deduce that ZnO continues being totally converted into zinc acetate by acetic acid vapor adsorption. Indeed, 12% (as 10% of 121%, the AcOH_ad_% of pure ZnO nps) was, within the limit of experimental error, exactly the difference in AcOH_ad_% values calculated between the pure supports (PVF and WP) and the corresponding inorganic–organic composites (PVF-ZnO and WP-ZnO, see Table 2). The presence of ZnO nanoparticles, which were able to irreversibly transform acetic acid into zinc acetate, could be considered a promising tool to counteract the “vinegar syndrome”, whereas the simple forms of adsorption (both chemi- and physi-) by the pure supports (WP and PVF) did not permit rendering the acetic acid inert. The presence of peaks ascribable to zinc acetate in the FTIR spectra acquired after the desorption test confirms this result (Figure 7).

Moreover, a higher efficacy was observed for PVF-ZnO when comparing this system with WP + ZnO (AcOH_ad_% of PVF + ZnO—27 ± 2 vs. WP + ZnO—14 ± 1%, Table 2). A possible explanation could be that the PVF xerogel’s porous structure succeeded in promoting the adsorption of acetic acid onto the surfaces of ZnO nanoparticles and the xerogel itself if compared to WP. By comparing the SEM micrographs of WP-ZnO (Figure 9A) and PVF (Figure S4)/PVF-ZnO (Figure 9C), a first, intuitive proof of this point could be derived, considering the higher porosities of the sponge. A further validation of this point could be achieved by increasing and quantifying the specific surface areas for both PVF and WP, which could provide a more accurate explanation of the superior acetic acid vapor adsorption achieved by PVF.

Concerning both WP-ZnO and PVF-ZnO, the weight increases after each step of the test were comparable, as previously highlighted, to those registered for pure nanoparticles (AcOH_convert._% of ZnO—120 ± 3%, Table 1). This is notable considering that the ZnO nanoparticles in the organic–inorganic nanocomposite represented only 10% by weight. Potential future developments of these new smart materials could involve exploring the possibility of loading a greater amount of ZnO nanoparticles within the PVF xerogels, in order to enhance the acetic acid adsorption capacity. However, it is not guaranteed that a greater loading would be feasible, nor that it would result in improved performance. Indeed, the nanoparticles tended to aggregate during the loading process in the xerogel, with a consequent decrease in the surface area, as evidenced by the SEM micrographs (Figure 9A,C). Nonetheless, we consider the current results promising (i.e., PVF xerogels loaded with 10% ZnO nanoparticles) in providing conservators with more user-friendly systems compared to pure nanoparticles, which could contaminate motion picture film cases.

On this basis, we decided to select some of these systems (ZnO nanoparticles, WP-ZnO and PVF-ZnO) in order to test their efficacy in inhibiting the deacetylation process in artificially degraded motion picture films.

### 3.2. Evaluation of the Performance of Composite Organic–Inorganic Systems in the Inhibition of the “Vinegar Syndrome”

To evaluate the efficacy of the developed smart organic–inorganic systems in the inhibition of the “vinegar syndrome” on real motion picture films, some CA-based frames were subjected to an artificial degradation protocol called ATM2.9 [20]. By exposing motion picture films to a saturated atmosphere of HCl vapors for 9 days, it was possible to trigger the deacetylation process (first step). After the elimination of the excess of HCl (second step), motion picture films artificially affected by the “vinegar syndrome” were stored for different times at 100% RH in order to promote the further evolution of the deacetylation reaction (third step). The meaning of this three-step protocol has been well-detailed and explained in reference [21]. In this phase, inhibitors (pure nanoparticles of ZnO, pure PVF and WP, WP-ZnO and PVF-ZnO) were put inside some of the chambers in order to evaluate the different behaviors of motion picture films stored with and without them, in terms of free acidity, acetyl content (evaluated through the Heterogeneous Saponification Method and FTIR-ATR Spectroscopy) and tensile strength [20]. 

In the free acidity (Figure 10A) and acetyl content data (Figure 10B), a clear trend can be observed: in the sample stored without any inhibitors, the deacetylation process was dramatically pushed until a lowering of the overall degree of substitution of the polymer (from cellulose diacetate to cellulose monoacetate [3,31]); a similar trend was also evident for RMPF stored in the presence of pure PVF and WP, for which the deacetylation was only slowed down. Pure PVF and WP only weakly adsorbed the acetic acid produced by the degraded films by trapping it inside their pores (in particular PVF), and creating hydrogen bonds without neutralizing it. Moreover, the availability of free hydroxyl groups inside both the structures (cellulose and polyvinylalcohol formaldehyde) probably promoted the adsorption of external moisture, contributing to the slowing down of the deacetylation process.

Concerning pure ZnO nanoparticles, their performances were intermediate between the untreated samples and the ones treated with inorganic–organic systems—the free acidity seemed stable around the initial value, without a sensible decrease, and, consequently, a slower but evident decrease was observed in the acetyl content. This could be because ZnO nanoparticles were stored inside a glass vial at the bottom of the chamber, and the acetic acid adsorption was limited by this feature. However, the storage of nanoparticles inside a proper container was mandatory in the view of easy handling for conservators and archivists, so as not to risk their dispersion and the contamination of the real motion picture films with the ZnO nanopowders.

On the other hand, both inorganic–organic systems made of WP and PVF doped with ZnO nanoparticles presented the best performance, being able to inhibit the artificially induced deacetylation process. The free acidity was reduced thanks to the conversion of the emitted acetic acid into zinc acetate, with the consequent stabilization of the acetyl content. Their capacity to adsorb both acetic acid and moisture was very effective in the inhibition of the induced “vinegar syndrome”, especially for PVF-ZnO xerogels. It is reasonable to assume that their porous structure promoted the interaction between acetic acid and ZnO nanoparticles, as confirmed by the preliminary acetic acid adsorption tests. The FTIR-ATR spectra (Figure 11) confirm these hypotheses.

The tensile strengths of all the RMPF (Figure 12) were altered by the ATM2.9 degradation protocol. The Young’s Modulus, calculated from the linear fitting of the σ/ε graphs corresponding to the range in which the material showed elastic behavior (0–20 N), was lower for all the samples (treated and untreated) than that registered for “time 0” of the third phase of the degradation protocol (P9_HCl5M). This was probably due to the exposure of all the samples to high moisture (RH 100%) for different times; high moisture has an impact not only on the deacetylation process, but also on the alteration of the emulsion layer, which is made of gelatine and soluble in water. As shown in a previous study [20], this degradation protocol did induce a partial loss of the emulsion layer, which surely influenced the tensile strength of the overall motion picture film, independently of the application of any inhibitor. Prefacing that, the Young’s Modulus value was lower for samples stored without any inhibitor than it was for the others, in accordance with previous results. Indeed, the subtraction of acetic acid—which acts as a catalyst not only for the deacetylation process but also for the depolymerization of the CA backbone [3]—by the inhibitors contributed to the conservation of the tensile strenght of the films. Comparing the Young’s Modulus values calculated for the RMPF stored with all the inhibitors, it was not possible to detect significant differences, due to the standard deviation.

## 4. Materials and Methods

### 4.1. Chemicals and Materials 

Nanorestore Plus®, made of calcium hydroxide, Ca(OH)_2_, nanoparticles dispersed in ethanol (10 g/L), was kindly provided by the Consorzio Interuniversitario per lo sviluppo dei Sistemi a Grande Interfase (CSGI Consortium), Italy; zinc oxide, ZnO, nanopowder (<100 nm particle size), formaldehyde, Triton X100, glutaraldehyde (solution in water 50 wt. %), hydrochloric acid 37%, glacial acetic acidwere purchased from Sigma Aldrich (St. Louis, MO, USA). Calcium carbonate (CaCO_3_, ≥99.0% powder) and cyclohexane (≥99.0%) were purchased from ACS reagent (St. Louis, MO, USA). Ethanol anhydrous denatured and sulfuric acid 96% were purchased from Carlo Erba Reagents (Cornaredo, Italy). Polyvinyl alcohol was purchased from Kuraray, Tokyo, Japan (Kuraray Poval 10–98). Sodium hydroxide (≥98.5% pellets anhydrous) was purchased from Acros Organics (Geel, Belgium). All the chemicals were used without further purification.

To obtain dry Ca(OH)_2_ nanoparticles, the Nanorestore Plus^®^ dispersion was fluxed with nitrogen for 6 h in order to promote the evaporation of ethanol. To obtain CaCO_3_ nanoparticles, Ca(OH)_2_ dispersion was left for twenty days under a hood to promote the carbonatation process. 

Whatman paper (WP, Maidstone, U.K.) sheets (55 mm Ø, Cat. No. 1001-055). 

The water used for all the procedures was purified by a Millipore Milli-Q Direct-Q^®^ & Direct-Q UV water purification system (Milano, Italy) water resistivity: 18,2 MΩ at 25 °C).

The real motion picture films (RMPF) used in the experiments came from a reel called “Vita di una pianta” (The life of a plant), an educational documentary of the “Sezione Cinescolastica Paravia” (Movies for schools Paravia), dated between the 1960s and 1970s. The film (support and emulsion) was produced by Ferrania (Savona, Italy), and it was made of cellulose diacetate, as demonstrated by the polarization test [37], the presence of the typical edge code “SAFETY” [1,4] and the FTIR-ATR analysis (Figure 10A, [20]). The thickness of the RMPF was 125 ± 3 µm.

### 4.2. PVF Xerogels Synthesis 

PVF sponges were synthesized following the procedures described in the patents US2,668,153 [38] and US2,609,347 [39], and papers [40,41,42,43].

The synthesis procedure could be summarized as follows: 0.5 g of Triton X100 was added to 20 g of a solution of PVA 10 wt. % in water, then the system was heated at 95 °C under vigorous stirring for 30 min. Then the proper amount of a formaldehyde solution in water (PVAOH_monomer_/formaldehyde molar ratio = 2.2) was added dropwise to the mixture, continuing to heat and stirring for at least 5 min, until the foam reached the maximum volume. The solution was left to cool to 60 °C under stirring, then 10 mL of 50% aqueous H_2_SO_4_ was added. The solution was poured into a mold and cured in an oven at 60 °C for 5 h. Then, the sample was washed with water at least five times to remove the residual solvents and unreacted products. The sponges were dried in an oven at 55–60 °C.

### 4.3. PVF-ZnO Composed Xerogels Synthesis

PVF + ZnO sponges were obtained by immersing the PVF sponge in a 10 g/L dispersion of ZnO nanoparticles in ethanol, previously sonicated for 30 min in an ultrasonic bath (Elmasonic S 30H, Elma Electronic, Fremont, CA, USA) with a power of 80 W, for 2 h under vacuum (15 mbar). The complete drying of the sponge was conducted by extracting the sponge from the dispersion and leaving it under vacuum for a further 2 h (W_dry_). The amount of nanoparticles loaded into the sponge was evaluated by weight, and was about 9.2 ± 0.9% of the initial weight of the sponge. 

### 4.4. WP-ZnO Synthesis

To obtain the WP-ZnO composite systems, a WP disk was immersed in a homogeneous dispersion of 5 g/L of ZnO nanoparticles in ethanol previously sonicated for 30 min in an ultrasonic bath (Elmasonic S 30H, Elma Electronic, USA) with a power of 80 W. Then the system was left to dry for 24 h to promote the complete evaporation of the dispersant. The amount of uploaded nanoparticles on the WP was evaluated by weight, and it was about 9 ± 1% of the initial weight of the WP (ca. 1.05 ± 0.3 mg/cm^2^). 

### 4.5. Acetic Acid Adsorption–Desorption Tests on Nanoparticles

To evaluate the capacities of the systems to adsorb acetic acid vapors, adsorption–desorption tests were performed on various nanoparticles (Ca(OH)_2_, CaCO_3_, ZnO) exposed to a saturated glacial acetic acid atmosphere. Nanopowders with nominally similar dimensions (<100 nm) were chosen. Also, a micropowder of CaCO_3_ (grain size in the order of 1–5 μm, see Figure S2E in the Supporting Information) was considered for comparison. All the systems were dried, their weights were registered (W_dry_, weight of dry sample), and they were put in sealed jars (130 mL) with a vial containing 8 mL of glacial acetic for six days (until saturation). Then, they were left to equilibrate for six more days at a controlled temperature and humidity (20 °C, RH 50%) and weighed (W_ad_).

The amount of adsorbed acetic acid after the adsorption test (AcOH_ad_%) was evaluated through gravimetric analysis, using the following equation:

(3) $${AcOH}_{ad}\%=(\frac{W_{ad}-W_{dry}}{W_{dry}})\cdot 100$$

where *W*_*dry*_ = weight of the dried sample, *W*_*ad*_ = weight (g) of the sample after the adsorption test.

To obtain further information about the nature of the interaction between the nanoparticles and the acetic acid vapors, desorption tests were performed [18]. Samples previously subjected to adsorption tests were put under vacuum (15 mbar) until they reached a stable weight (about 48 h). Assuming that the non-desorbed acetic acid completely reacted with the nanoparticles, converting them into acetate salts, the amount of such entirely converted acetic acid (AcOH_convert._%) after the equilibration period was evaluated according to the following equation:

(4) $${AcOH}_{convert.}\%=(\frac{W_{des}-W_{dry}}{W_{dry}})\cdot 100$$

where *W*_*dry*_ = weight (g) of the dried sample, *W*_*des*_ = weight (g) of the sample after the desorption test. 

The residual adsorbed acetic acid (AcOH_resid._%) after the adsorption period was calculated as:

(5) $${AcOH}_{resid.}\%={AcOH}_{ad}\%-{AcOH}_{convert.}\%$$

For each test, three samples were prepared, and the average values and the corresponding standard deviation are reported below.

For pure nanoparticles, FTIR spectra, XRD diffractograms, SEM micrographs and TGA profiles were acquired before and after the adsorption test with the following methodologies.

Fourier Transform Infrared (FTIR) Spectroscopy. FTIR measurements were performed using a BioRad FTS-40 spectrometer (Bio-Rad Laboratories, Hercules, CA, USA) in the range 4000–400 cm^−1^. The spectra were averages of 64 scans recorded in transmittance mode with 2 cm^−1^ resolution. KBr pellets were prepared by finely grinding and mixing a few milligrams of sample and 200 mg of pure KBr.

X-ray diffraction (XRD). Powder X-ray diffraction (XRD) analyses were carried out at the CRIST Centre of the University of Florence (Florence, Italy). A Bruker D8 Advance diffractometer (USA) equipped with Cu Kα radiation and a Lynx Eye detector (Bruker, Billerica, MA, USA) was used, operating in θ–2θ Bragg–Brentano geometry at 40 kV and 40 mA, in the range of 4–55/65° with a step size of 0.04° and a count time of 1 s. 

Thermogravimetric Analysis (TGA). Tests were performed in a nitrogen atmosphere at a heating rate of 10 °C/min over a temperature range of 25–500 °C, with an initial sample weight of approximately 5 mg using an SDT 650 thermal analyzer (TA, New Castle, DE, USA).

Field-Emission Scanning Electron Microscopy (FE-SEM). SEM micrographs were collected by means of a Zeiss Sigma FE-SEM instrument (Italy), operating in high vacuum mode with an acceleration potential of 2–3 kV. It was equipped with a GEMINI column and an In-Lens detector. Nanoparticles of Ca(OH)_2_, CaCO_3_, and ZnO were dispersed in ethanol, while the corresponding acetate salts in cyclohexane (0.1 g/L) were sonicated with a Digital Sonifer 250, Branson (Danbury, CT, USA), equipped with a microtip, for 5 min at 30% amplitude. After applying a few drops of dispersions onto the stub, and after the evaporation of the dispersant, the metallization was performed using gold vapor under vacuum.

For Ca(OH)_2_, CaCO_3_, CaCO_3__AcOH, ZnO and ZnO_AcOH, a size distribution was calculated from micrographs of 10.000 K magnitude using Image J software (v. 1.54). Nanoparticles were approximated into rounded systems, and the calculation was performed on three micrographs for each sample. The average radius and the corresponding standard deviation are reported.

### 4.6. Artificial Induction and Evolution of the “Vinegar Syndrome” on Motion Picture Films and Their Characterization

The performances of ZnO pure nanoparticles, and WP-ZnO and PVF-ZnO xerogels, in the inhibition of the “vinegar syndrome” in real motion picture films (RMPF) on which the deacetylation process had been artificially induced was evaluated. We chose to apply the ATM2.9 degradation protocol reported in [20]. The RMPF were cut into fragments of 50 × 16 mm (total amount for each chamber, ca. 1 g) and hung at the top of the chamber with a Teflon wire, spaced out with glass marbles with a diameter of about 2 mm to avoid their mutual contact during the storage. Pure WP and WP-ZnO sheets were put in between the RMPF fragments spaced out with glass marbles, and pure PVF and PVF-ZnO xerogels were put at the bottoms of the chambers at about 0.5 cm from the motion picture films, not in direct contact with them. Pure ZnO nanoparticles were put in a glass vial at the bottom of the chamber.

The methods and techniques used to monitor the evolution of the deacetylation process are reported in a previously published paper [20]. A resume of the analyzed samples is reported in Table 3.

## 5. Conclusions

In this study, the possibility of using innovative inorganic–organic nanocomposite systems based on inorganic nanoparticles for the inhibition of the “vinegar syndrome” in cellulose acetate-based motion picture films was evaluated. 

First of all, the capacities of various nanoparticles (Ca(OH)_2_, ZnO and CaCO_3_) to convert acetic acid vapors into the corresponding acetate salt were confirmed through FTIR, XRD, TGA, SEM and gravimetric analyses. All the systems were revealed to be effective for this purpose. Then, the most effective and stable of these species (ZnO) was used to dope two different organic systems made of Whatman paper (WP-ZnO) and porous xerogels of PVF (PVF-ZnO). The intent was to propose effective systems, able to adsorb and neutralize the acetic acid emitted by CA-based motion picture films, and consequently inhibit the “vinegar syndrome”, and to provide easy-to-handle objects for conservators and archivists. Both WP-ZnO and PVF-ZnO could be easily inserted inside the cases in which films are stored without the risk of dispersing nanoparticles. Moreover, their shape can be easily adapted according to the needs. To prove the capacity of these systems to inhibit the deacetylation process and its consequences, CA-based real motion picture films were subjected to an artificial degradation process with and without our proposed inhibitors. From the comparison of samples stored with and without the systems and their characterization in terms of free acidity, acetyl content, and tensile strength, both WP-ZnO and PVF-ZnO were found to be effective in the inhibition of the “vinegar syndrome”. Indeed, the decrease in the free acidity due to the capacity of these inorganic–organic systems to adsorb moisture and neutralize acetic acid, as seen from the preliminary adsorption tests of acetic acid vapors, ensured the stabilization of the acetyl content of the film. Also, the decrease in tensile strength was significantly slowed by the presence of the inhibitors, because of the removal of the catalyst for the depolymerization reaction.

Another interesting point regards the effectiveness of these systems even when not in direct contact with the film. This is an important advantage in terms of both ease of application (it is not necessary to ensure contact between the inhibitor and the film for the entire length of the film) and the risk of altering the visual integrity of the object. Future experiments on films “naturally” affected by the “vinegar syndrome” will be crucial to assess the performance of these systems in real-world scenarios. Additionally, optimizing the methods for loading nanoparticles into both paper and xerogels should be considered. The use of more sustainable xerogels as an alternative to PVF could also be recommended for future applications. Nonetheless, the results presented in this paper provide a promising proof of concept for the efficacy of inorganic nanoparticles, and represent a crucial starting point for evaluating these inorganic–organic nanocomposite systems as innovative inhibitors of the “vinegar syndrome” in CA-based motion picture films.

## Figures and Tables

**Table 1:** Adsorbed acetic acid values calculated after the adsorption test (AcOH_ad_%) and after the desorption test (AcOH_convert_.%: acetic acid entirely converted into acetate salts; AcOH_resid_.%: residual adsorbed acetic acid not converted into acetate) and the theoretical increase in weight expected from the conversion of all the starting nanoparticles to the corresponding acetate salt (AcOH_T_%) for Ca(OH)_2_, CaCO_3_/Ca(OH)_2_, ZnO, nanoparticles, and CaCO_3_ micropowder. For the mixture CaCO_3_/Ca(OH)_2_, the theoretical value AcOH_T_% of 100% conversion into acetate cannot be determined.

Scheme	AcOH_ad_% *	AcOH_convert._% °	AcOH_resid._% §	AcOH_T_% @
Ca(OH)_2_	113 ± 1	111 ± 4	2 ± 3	113
CaCO_3_/Ca(OH)_2_	64 ± 2	62 ± 5	2 ± 5	/**
ZnO	121 ± 3	120 ± 6	1 ± 2	125
CaCO_3_ micro	10 ± 1	9 ± 1	1 ± 2	58

@ Equation (1), * Equation (3), ° Equation (4), § Equation (5), and ** for pure CaCO_3_; AcOH_T_ would be 58%.

**Table 2:** Percentage of acetic acid adsorbed in weight calculated after the adsorption test (AcOH_ad_%) and after the desorption test (AcOH_covert._% and AcOH_resid._%) for PVF + ZnO, pure PVF, WP+ZnO, pure WP and ZnO nanoparticles.

Sample	AcOH_ad_% *	AcOH_convert._% °	AcOH_resid._% §
PVF + ZnO	27 ± 2	20 ± 2	7 ± 2
PVF	17 ± 1	6 ± 1	11 ± 1
WP + ZnO	17 ± 1	14 ± 1	3 ± 1
WP	6.7 ± 0.7	3.5 ± 0.4	3.2 ± 0.5
ZnO nps	121 ± 3	120 ± 3	1 ± 3

* Equation (3), ° Equation (4), § Equation (5).

**Table 3:** Resume of all the samples used to evaluate the effects of our treatment in inhibiting the artificially induced deacetylation process (the labels not yet described refer to: P = duration of the third degradation step (apart from P_HCL5M, for which it is equal to first degradation step), ATM2.9 = degradation protocol reported in reference [21], NT = not treated, WP = Whatman paper, nps = nanaparticles).

Sample	Duration of the First Degradation Step (days)	Duration of the Second Degradation Step (days)	Duration of the Third Degradation Step (days)	Treatment
P9_HCl5M	9	1	/	/
P12_ATM2.9_NT	9	1	12	/
P24_ATM2.9_NT	9	1	24	/
P36_ATM2.9_NT	9	1	36	/
P48_ATM2.9_NT	9	1	48	/
P12_ATM2.9_WP	9	1	12	Pure WP (total weight 0.45 g)
P24_ATM2.9_WP	9	1	24	
P36_ATM2.9_WP	9	1	36	
P48_ATM2.9_WP	9	1	48	
P12_ATM2.9_WPZnO	9	1	12	WP uploaded with ZnO nps (total weight: 0.5 g)
P24_ATM2.9_WPZnO	9	1	24	
P36_ATM2.9_WPZnO	9	1	36	
P48_ATM2.9_WPZnO	9	1	48	
P12_ATM2.9_PVF	9	1	12	PVF sponge at the bottom of the jar (0.3 g)
P24_ATM2.9_PVF	9	1	24	
P36_ATM2.9_PVF	9	1	36	
P48_ATM2.9_PVF	9	1	48	
P12_ATM2.9_PVF+nps	9	1	12	PVF sponge uploaded with ZnO nps at the bottom of the jar (0.3 g)
P24_ATM2.9_PVF+nps	9	1	24	
P36_ATM2.9_PVF+nps	9	1	36	
P48_ATM2.9_PVF+nps	9	1	48	

## Data Availability

Data are contained within the article.

## Supplementary Materials

The following supporting information can be downloaded at: https://www.mdpi.com/article/10.3390/molecules30061348/s1, Figure S1: FTIR spectra of Ca(OH)_2_ (A), ZnO (B) and CaCO_3_ (C) nanoparticles before (black) and after (red) the acetic acid adsorption test; Figure S2. SEM micrographs acquired for Ca(OH)_2_ before (A) and after (B) the adsorption test, CaCO_3_ nanoparticles before (C) and after (D) the adsorption test, CaCO_3_ micropowder before (E) and after (F) the adsorption test; Figure S3. TGA (solid line) and DTG (dashed line) profiles of PVF (black) and PVF-ZnO (red); Figure S4. FE-SEM micrographs acquired of the pure PVF sponge (A) and the PVF+ZnO sponge (B) with 5.00kx magnitude; Figure S5. Microtomographies of PVF (A,B) and PVF-ZnO (C,D) in false-colors to reveal the density variation due to the presence of ZnO nanoparticles [18,22,30,44,45].
