# Supplementary material for: Adsorption of Acetic Acid Vapors by Inorganic–Organic Nano Materials: Implications for the Inhibition of the “Vinegar Syndrome” in 20th Century Motion Picture Films
Source: Molecules. 2025 Mar 17;30(6):1348. doi: 10.3390/molecules30061348 (PMC11945910; doi:10.3390/molecules30061348)
Supplement: Supplementary file 1 [file molecules-30-01348-s001.zip › molecules-3498506-supplementary.pdf]

Supporting Information

Adsorption of Acetic Acid Vapors by Inorganic–Organic Nano Materials: Implications for the Inhibition of the “Vinegar Syndrome” in 20th Century Motion Picture Films

Francesca Porpora [1], Lorenzo Lisi [1], Emiliano Carretti [1,2,*], Carlotta D’Aleo [1], Marianna De Sanctis [3], Samuele Baldini [1] and Luigi Dei [1,*]

[1] Department of Chemistry “Ugo Schiff” & CSGI Consortium, University of Florence, Via Della Lastruccia 3-13, 50019 Sesto Fiorentino, Italy; francesca.porpora@unifi.it (F.P.); lore.lisi31@gmail.com (L.L.);
carlotta.daleo@edu.unifi.it (C.D.); samuele.baldini@unifi.it (S.B.)

[2] Centro Nazionale Delle Richerche—Istituto Nazionale di Ottica (CNR-INO), Largo E. Fermi 6, 50125 Firenze, Italy

[3] Film Restoration Laboratory “L’Immagine Ritrovata”, Via Riva di Reno 72, 40122 Bologna, Italy; marianna.desanctis@immagineritrovata.it

* Correspondence: emiliano.carretti@unifi.it (E.C.); luigi.dei@unifi.it (L.D.)

1. *Acetic acid adsorption-desorption tests on nanoparticles.*

1.1 FTIR spectra

FTIR spectra of $Ca(OH)_2$, ZnO and $CaCO_3$ before and after the adsorption were collected.
For both the FTIR spectra of $Ca(OH)_2$ (Fig. SI1A), peaks associated with the stretching of the O–H bond of the hydroxide at 3646 and 3696 cm^{-1} were present. In the spectrum registered after the adsorption test, there were some signals that could be attributed to the presence of acetate [1,2] (Fig. SI1A), as follows:

1. The peaks in the region between 3000 and 2850 cm^{-1} due to the CH_3 stretching were ascribable to calcium acetate;
2. The strong band at 1540 cm^{-1} was due to C–O antisymmetric stretching vibrations, and the one at 1443 cm^{-1} was attributed to the symmetric stretching vibration of the C–O bond;
3. The out-of-plane stretching vibration of the methyl group that was split into two peaks at 1057 and 1022 cm^{-1};
4. The peak at 946 cm^{-1} was attributable to the n(C–C) stretching vibration of the acetate anion;
5. The two peaks at 636 and 616 cm^{-1} were due to the out-of-plane stretching vibration of the O–C–O fragment of the acetate anion.

Also, for ZnO (Fig. SI1B), its partial conversion to zinc acetate was evident, as indicated by [3,4]:

1. The peaks associated with the COO- asymmetric and symmetric stretching vibrations modes at 1540 and 1431 cm^{-1}, respectively;
2. The minor peak at 1380 cm^{-1} was related to the symmetric stretching mode of the CH_3 group.

The $CaCO_3$ spectrum after the adsorption test (Fig. SI1C) showed promising results, too. Also, in this case, peaks associated with the formation of the acetate salt were present [5]—characteristic peaks at 1529 and 1440 cm^{-1}, at 1354 cm^{-1}, and two peaks at 1054 and 1022 cm^{-1} were visible.
From all these spectra, it was possible to confirm that the increase in weight monitored through gravimetry tests was actually due to the conversion of the initial hydroxide/oxide/carbonate into its corresponding acetate salt. However, it was not possible to exclude that partial carbonatation and moisture retention also influenced this increase.

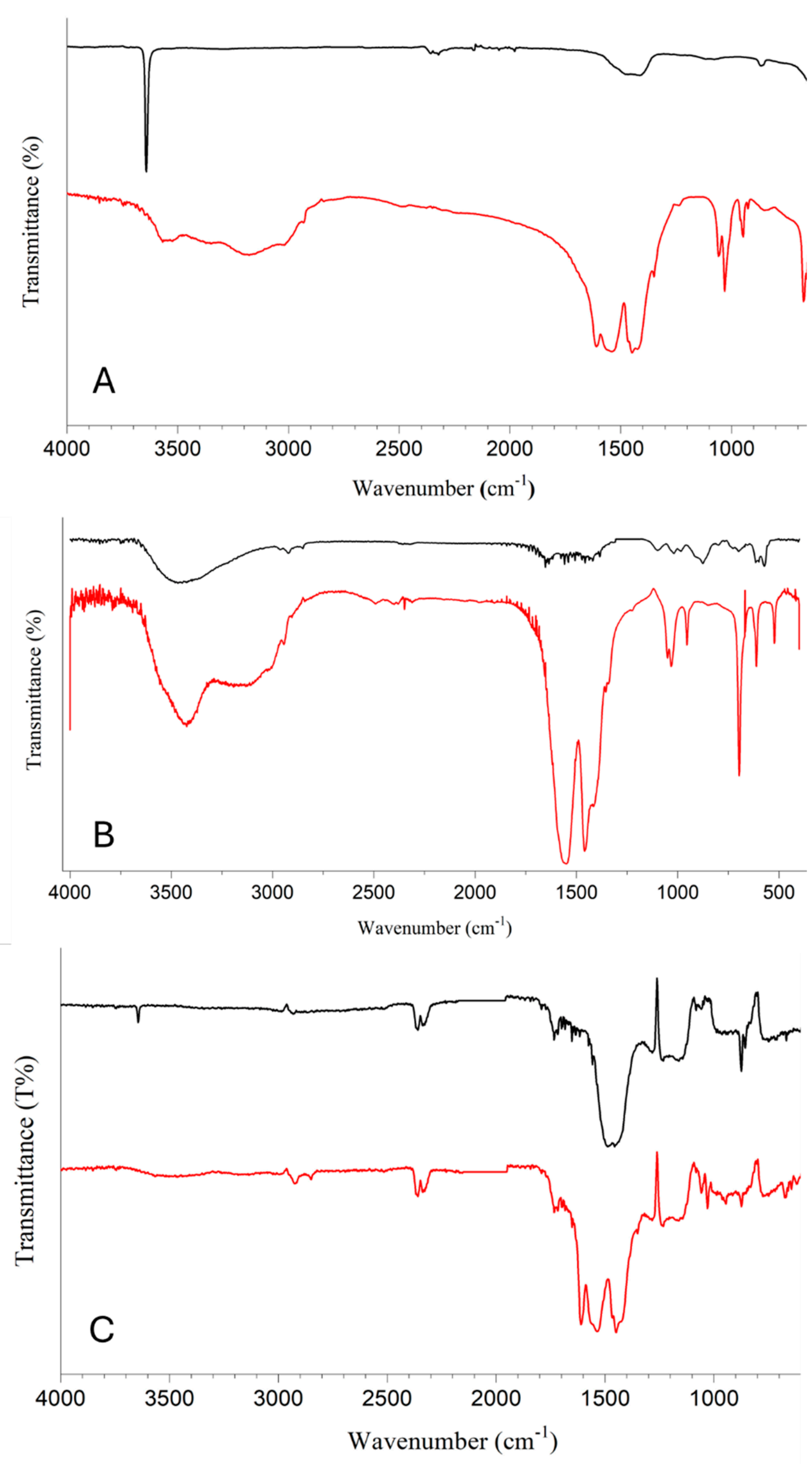

Figure S1. FTIR spectra of $Ca(OH)_2$ (A), ZnO (B) and $CaCO_3$ (C) nanoparticles before (black) and after (red) the acetic acid adsorption test.

1.2 *SEM micrographs*

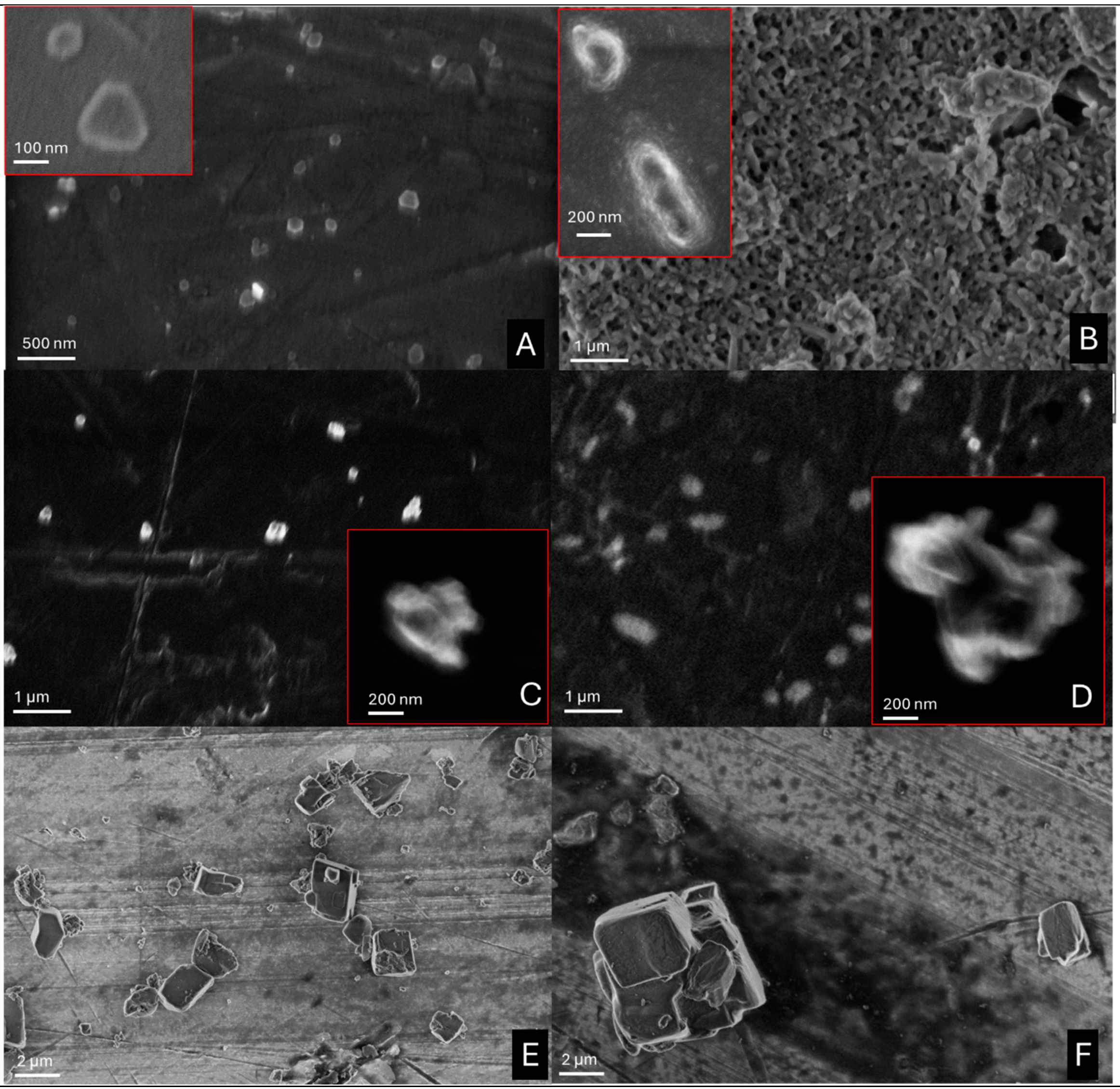

Figure S2. SEM micrographs acquired for $Ca(OH)_2$ before (A) and after (B) the adsorption test, $CaCO_3$ nanoparticles before (C) and after (D) the adsorption test, $CaCO_3$ micropowder before (E) and after (F) the adsorption test.

2. ***Characterization of PVF-ZnO system***

2.1 *Material and Methods*

Thermogravimetric analysis (TGA). Tests were performed as reported in Paragraph 4.5.

Field-Emission Scanning Electron Microscopy (FE-SEM). SEM micrographs were collected by means of a Zeiss Sigma FE-SEM instrument, operating in high vacuum mode with an acceleration potential of 2 kV. It was equipped with a GEMINI column and In-Lens detector. *X ray microtomography (μ-TOM).* X-ray microtomography measurements were carried out with a Skyscan 1172 high-resolution MicoCT system at CRIST Centre, University of Florence (Italy) on a sample of ~1x0.5x0.25 cm. The X-rays tube equipped with a tungsten anode was operated at 100 kV and 100 μA. By placing the sample between the X-ray source and the CCD detector, 2D X-ray images were captured over a 180-degree rotating sample with a slice-to-slice rotation angle of 0.3. The spatial resolution of the image was kept in a range of 4 μm in terms of pixel size. The 3D image was reconstructed from the projections using the Nrecon software (Bruker μ-CT 1.6.10.2). After reconstruction, the image was analyzed to obtain information on the sponge structure, such as the pore

size distribution, through the CTAnalyser software (Bruker μ-CT 1.18.8.0). A 3D representation in false-color was realized by the CTVox software (Bruker μ-CT 3.3.0).

2.2 Results

2.2.1 TGA

In the TGA/DTG profile of PVF-ZnO (Figure SI3), the first weight loss of 2.6% between 35 and 250 °C is ascribable to the loss of moisture in the PVF network (i.e., bulk water and strongly bound water), and the second weight loss of 78.1% between 250 and 450 °C (which corresponds to the two peaks at 330 and 419 °C in the DTG curve) could be associated with the degradation of PVF (in pure PVF, a weight loss of 91 wt. % was detected in the same range, as indicated by the peak at 392 °C, Figure SI3). The presence of two peaks in the DTG profile of PVF-ZnO was nto clear, while in the DTG profile of PVF, only one was detected. To better understand this point further, SAXS and rheological analysis are needed. The residual 10% of weight was probably due to the ZnO nanoparticles inside the xerogel; the thermal decomposition of ZnO was not detectable in this temperature range because its degradation occurs above 500 °C.

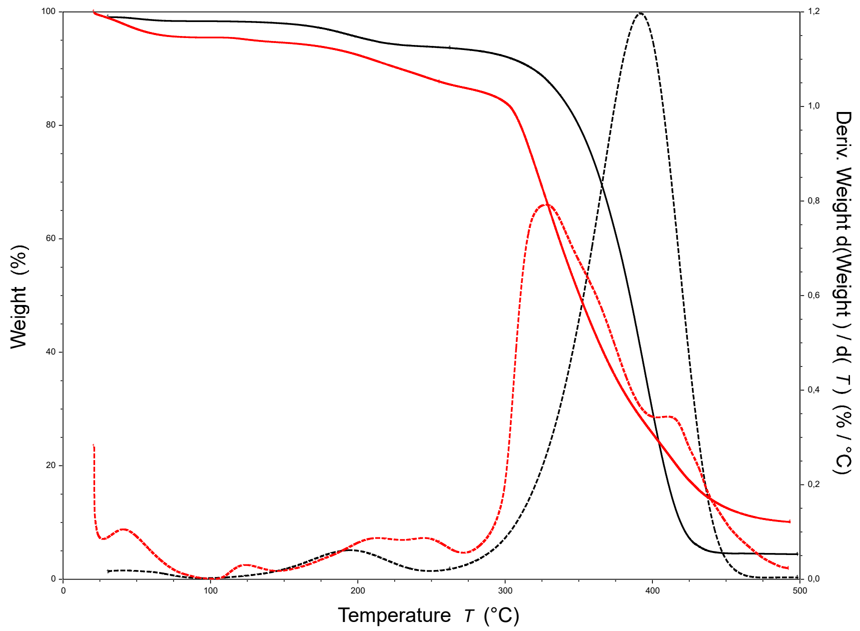

Figure. S3. TGA (solid line) and DTG (dashed line) profiles of PVF (black) and PVF-ZnO (red).

2.2.2. FE-SEM

Before analyzing the adsorption capacity of glacial acetic acid vapors by these systems, their structures were analyzed through FE-SEM. In Figure SI4, the presence of nanoparticles on the surface of a PVF sponge is clear; while the sample of pure PVF (Figure SI4A) has a clean surface only interrupted by porosities (with diameters from 2 μm to more than 300 μm), in the PVF+ZnO sponge, ZnO nanoparticles are visible (Figure. SI4B). It is possible to see the presence of both isolated nanoparticles, which preserved their size and structure, as well as bigger aggregates of several microns.

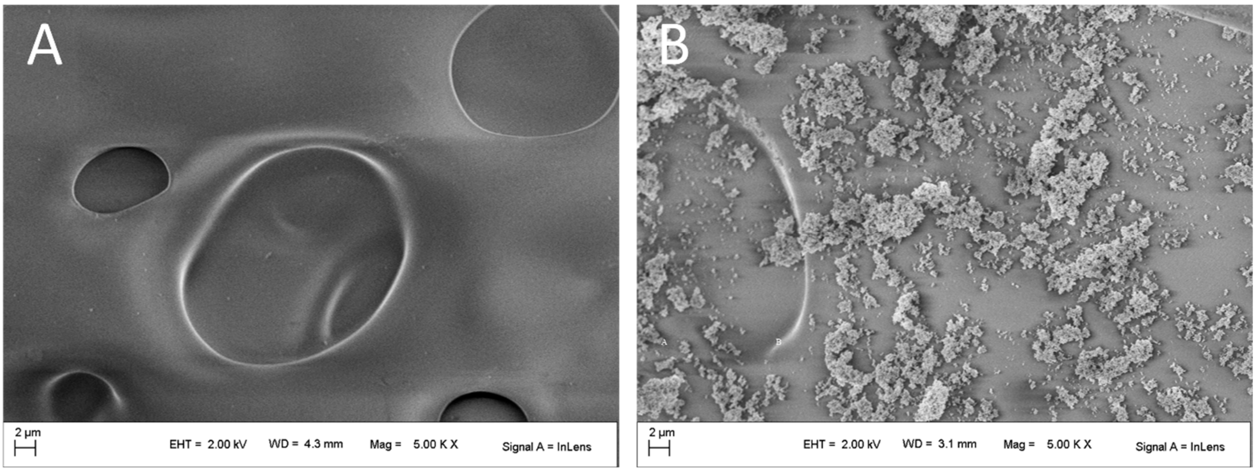

Figure. S4. FE-SEM micrographs acquired of the pure PVF sponge (A) and the PVF+ZnO sponge (B) with 5.00kx magnitude.

2.2.3 μ-TOM

The microtomographies acquired for PVF (Fig. SI5A) and PVF-ZnO (Fig. SI5B) show highly porous structures.

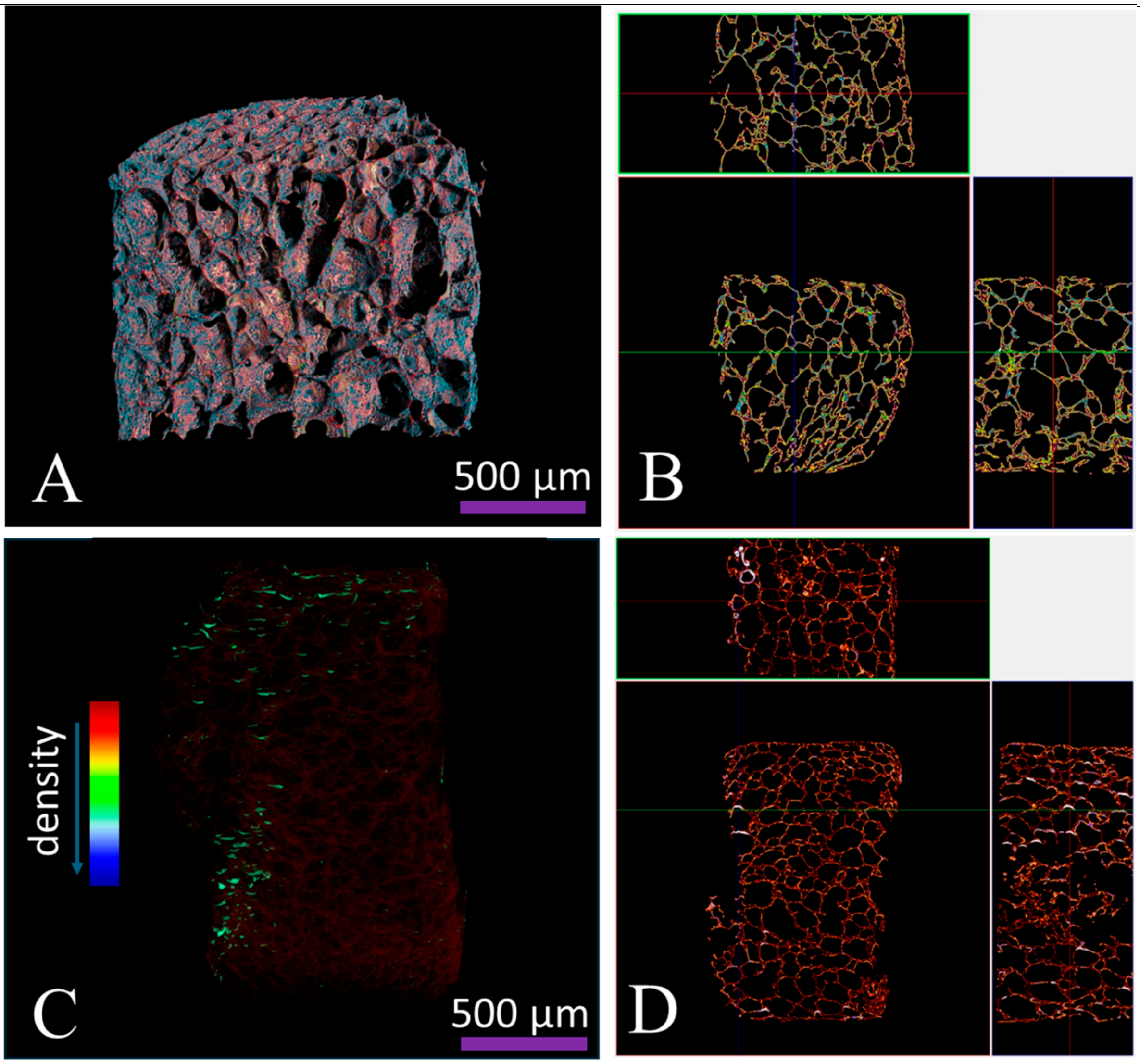

Figure S5. Microtomographies of PVF (A-B) and PVF-ZnO (C-D) in false-colors to reveal the density variation due to the presence of ZnO nanoparticles.

References

1. Thongkam, M.; Saelim, J.; Boonchom, B.; Seesanong, S.; Chaiseeda, K.; Laohavisuti, N.; Bunya-Atichart, K.; Boonmee, W.; Taemchuay, D. Research Article Simple and Rapid Synthesis of Calcium Acetate from Scallop Shells to Reduce Environmental Issues. **2021**, doi:10.1155/2021/6450289.

2. Pemberton, A.T.; Brandon, D.; King, D.A. Integrated TGA, FTIR, and Computational Laboratory Experiment. **2018**, doi:10.1021/acs.jchemed.8b00607.

3. Yin, H.; Casey, P.S. ZnO Nanorod Composite with Quenched Photoactivity for UV Protection Application. *Mater Lett* **2014**, *121*, 8–11, doi:10.1016/j.matlet.2014.01.103.

4. Zuliani, A.; Bandelli, D.; Chelazzi, D.; Giorgi, R.; Baglioni, P. Environmentally Friendly ZnO/Castor Oil Polyurethane Composites for the Gas-Phase Adsorption of Acetic Acid. *J Colloid Interface Sci* **2022**, *614*, 451–459, doi:10.1016/j.jcis.2022.01.123.

5. Pang, S.F.; Wu, C.Q.; Zhang, Q.N.; Zhang, Y.H. The Structural Evolution of Magnesium Acetate Complex in Aerosols by FTIR-ATR Spectra. *J Mol Struct* **2015**, *1087*, 46–50, doi:10.1016/j.molstruc.2015.01.034.
